# Isolation of Small Extracellular Vesicles (sEVs) from the Apoplastic Wash Fluid of *Nicotiana benthamiana* Leaves

**DOI:** 10.1002/cpz1.70026

**Published:** 2024-11-05

**Authors:** Mahmoud K. Eldahshoury, Konstantina Katsarou, Joshua T. Farley, Kriton Kalantidis, Carine de Marcos Lousa

**Affiliations:** ^1^ Biomedical Sciences, School of Health Leeds Beckett University Leeds UK; ^2^ Institute of Molecular Biology and Biotechnology Foundation for Research and Technology‐Hellas Heraklion Crete Greece; ^3^ Department of Biology, University of Crete Voutes University Campus Heraklion Crete Greece; ^4^ Centre for Plant sciences University of Leeds Leeds UK

**Keywords:** apoplastic fluid, EV, *Nicotiana benthamiana*, PEN1, plant, small extracellular vesicles (sEVs), TET8

## Abstract

Extracellular vesicles (EVs) are small membranous vesicles secreted by cells into their surrounding extracellular environment. Similar to mammalian EVs, plant EVs have emerged as essential mediators of intercellular communication in plants that facilitate the transfer of biological material between cells. They also play essential roles in diverse physiological processes including stress responses, developmental regulation, and defense mechanisms against pathogens. In addition, plant EVs have demonstrated promising health benefits as well as potential therapeutic effects in mammalian health. Despite the plethora of potential applications using plant EVs, their isolation and characterization remains challenging. In contrast to mammalian EVs, which benefit from more standardized isolation protocols, methods for isolating plant EVs can vary depending on the starting material used, resulting in diverse levels of purity and composition. Additionally, the field suffers from the lack of plant EV markers. Nevertheless, three main EV subclasses have been described from leaf apoplasts: tetraspanin 8 positive (TET8), penetration‐1‐positive (PEN1), and EXPO vesicles derived from exocyst‐positive organelles (EXPO). Here, we present an optimized protocol for the isolation and enrichment of small EVs (sEVs; <200 nm) from the apoplastic fluid from *Nicotiana benthamiana* leaves by ultracentrifugation. We analyze the preparation through transmitted electron microscopy (TEM), nanoparticle tracking analysis (NTA), and western blotting. We believe this method will establish a basic protocol for the isolation of EVs from *N. benthamiana* leaves, and we discuss technical considerations to be evaluated by each researcher working towards improving their plant sEV preparations. © 2024 The Author(s). Current Protocols published by Wiley Periodicals LLC.

**Basic Protocol**: Isolation and enrichment of small extracellular vesicles (sEVs) from the apoplastic fluid of *Nicotiana benthamiana* leaves

## INTRODUCTION

Extracellular vesicles (EVs) are now established as crucial mediators of intercellular communication in various biological systems. These small membrane‐bound vesicles are believed to facilitate the exchange of biomolecules, such as proteins, nucleic acids, and lipids, in various living organisms including plants (di Bella et al., 2022; Lian et al., 2022). Research on plant EVs has gained considerable attention in recent years. Exploration of EVs in botanical systems not only enhances our understanding of plant cellular processes but also holds the promise of applications in agriculture and biomedicine (Orefice et al., 2023, Yang et al., 2023).

*Nicotiana benthamiana*, a member of the Solanaceae family and a popular model organism in the study of plant biology and host‐pathogen interactions, has been of interest to the scientific community for a few reasons. Its rapid growth allows the generation of a substantial amount of biological material in a reasonable time (3‐4 weeks after germination under optimal conditions; Goodin et al., 2008; Ranawaka et al., 2023). In addition, this model plant exhibits a high susceptibility to a wide range of viruses and other plant pathogens, making it ideal for studying plant‐pathogen interactions (Goodin et al., 2008); furthermore, *N. benthamiana* is an ideal system in which to transiently express *in planta* any gene of interest via a method known as agroinfiltration (Goodin et al., 2008; Sparkes et al., 2006). Finally, *N. benthamiana* has attracted pharmaceutical interest due to its scaling‐up capacity for the production of therapeutic material (Song et al., 2022). These advantages have established this species as a major plant model organisms alongside *Arabidopsis thaliana*. Nevertheless, despite extensive research on *N. benthamiana*, the presence and functional significance of EVs in this plant species remain little understood.

Although few protocols have been published for the isolation and purification of small EVs (sEVs) from *A. thaliana* (Chen et al., 2022; Rutter & Innes, 2017), there have been only limited reports of sEV isolation and purification from *N. benthamiana* leaf apoplastic fluid (Mohaved et al., 2019; Wang et al., 2024; Zhang et al., 2020). This article describes an optimized method to extract sEVs from the apoplastic fluid of *N. benthamiana* leaves. The protocol incorporates a minimal number of centrifugation steps followed by pelleting of the sEVs by ultracentrifugation at 100,000 × *g*. Although this approach somewhat lengthens the procedure, the extra steps enhance the clarity and purity of the EV preparation significantly, as demonstrated by various techniques such as TEM and NTA. We also validate the presence of PEN1‐ and TET8‐positive vesicles using western blot analysis. This adapted protocol for the isolation and enrichment of sEVs (<200 nm; Welsh et al., 2024) from the apoplastic fluid of *N. benthamiana* leaves serves as a foundation for EV purification and can be followed by other methods such as density gradients or immunoprecipitation (Chen et al., 2022), depending on the application's specific requirements.

## ISOLATION AND ENRICHMENT OF SMALL EXTRACELLULAR VESICLES (sEVs) FROM THE APOPLASTIC FLUID OF *Nicotiana benthamiana* LEAVES

**Figure 1:**
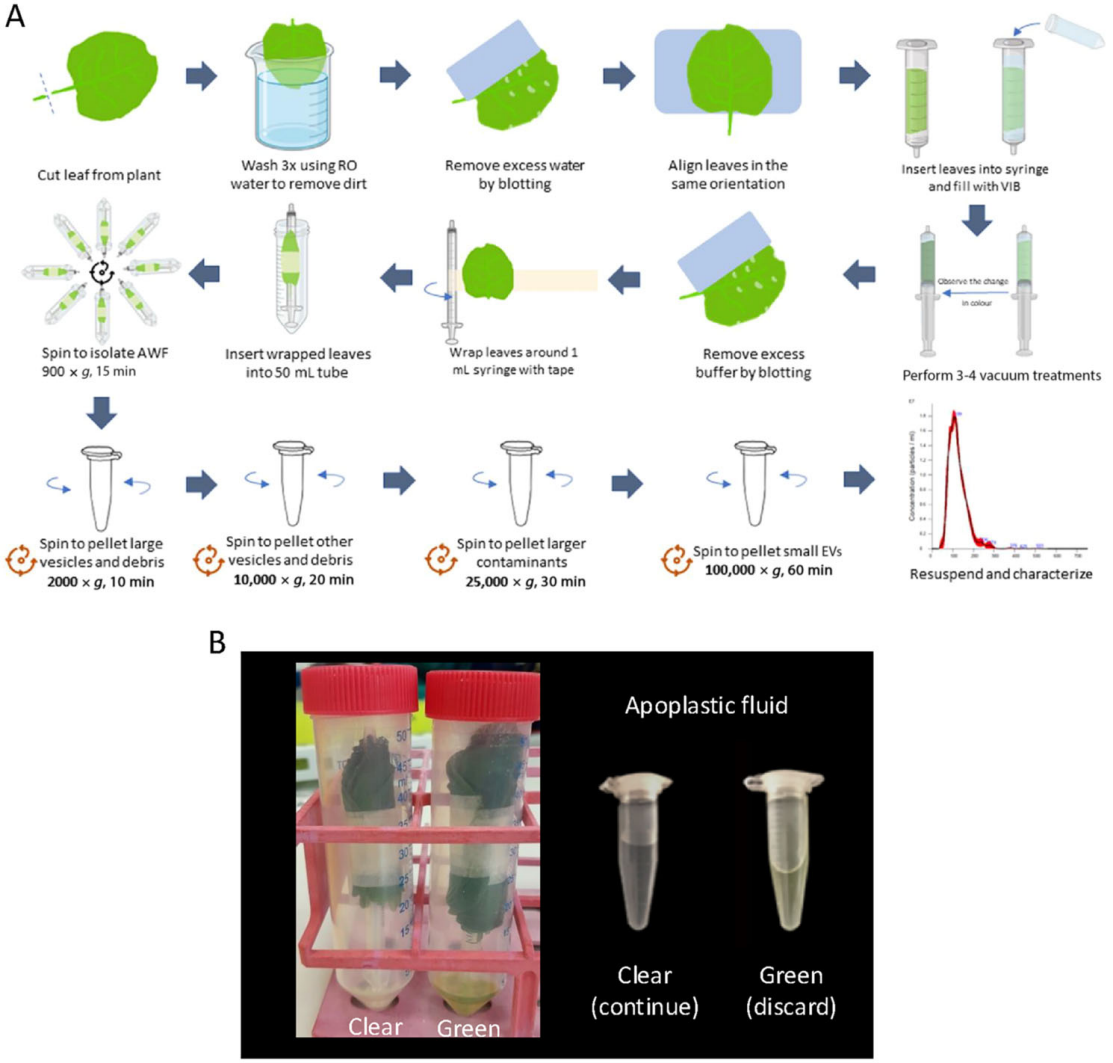
General workflow for basic protocol of sEV extractions from *N. benthamiana* leaves. (**A**) Graphical workflow describing the basic steps, including collection of apoplastic wash fluid through three low‐speed centrifugation steps—at 2000 × *g* for 10 min, 10,000 × *g* for 20 min, and 25,000 × *g* for 30 min—and a final spin at 100,000 × *g* for 60 min. The pellet of sEVs is resuspended carefully in buffer and analyzed by NTA, TEM, and/or western blotting. The remaining suspension is aliquoted and stored at an appropriate temperature for future density gradients or immunoprecipitation. (**B**) Example of tubes showing the difference between clear (left) and green (right) AWF. The green color indicates contamination by intracellular organelles, and these tubes should be discarded and the procedure should start over.

This protocol describes the purification of sEVs from *N. benthamiana* leaves. The initial stage for isolating the apoplastic wash fluid (AWF) from *N. benthamiana* leaves is conducted in accordance with a protocol developed to isolate AWF from *Phaseolus vulgaris* leaves (O'Leary et al., 2014). The second part details the enrichment of sEVs from AWF and was adapted from a procedure for EV enrichment from *A. thaliana* AWF (Chen et al., 2022; Huang et al., 2021). The filtration step was removed, and adjustments to the centrifugation steps were made, including the addition of a 25,000 × *g* centrifugation step between the 10,000 × *g* and 100,000 × *g* steps. These modifications result in a superior‐quality preparation compared to conventional protocols (Fig. 1). The use of swinging‐bucket or fixed‐angle rotors at the last stage of ultracentrifugation was also evaluated and is discussed in the Critical Parameters and Troubleshooting section below.

### Materials

*Nicotiana benthamiana* plants
Distilled water
Vesicle infiltration buffer (VIB): 20 mM 2‐morpholinoethanesulfonic acid (MES) hydrate/2 mM sodium chloride (CaCl_2_)/0.1 M sodium chloride (NaCl), pH 6 (pH adjusted with 1 N NaOH) (see Critical Parameters)
Phosphate‐buffered saline (PBS; Gibco, 10010023), freshly filtered (see Critical Parameters; optional)
Protease inhibitor (Sigma, P9599; optional)

100‐ or 60‐ml syringes
1‐ml syringes
Blue roll or paper towels
50‐ml conical tubes (Falcon type)Scalpel and scissors
Tape
Ultracentrifugation tubes (Beckman Coulter)
Refrigerated centrifuge for low‐speed centrifugation (2000 and 10,000 × *g*)
Ultracentrifuge (Beckman Coulter)
Ultracentrifugation rotors (Beckman Coulter: 70i Ti, SW32Ti, TLA‐110, MLA‐55)
Malvern NS300 Nanoparticle tracker (Nanosight‐ NTA)

### Preparation of N. benthamiana leaves for sEV isolation

Before starting the protocol, turn on centrifuges and set them all to 4°C.

1 Harvest the desired leaves from the plants, and remove petioles using either scissors or a scalpel. At least 50 g of tissue is needed to prepare sufficient sEVs.
  Plants can be grown either in a growth chamber or in a greenhouse. If grown in a growth chamber, use plants with five leaves, while under greenhouse conditions, older plants with five to eight leaves are preferrable. Plants should not reach the flowering stage. Using a specific amount of tissue rather than a specific number of leaves is recommended to ensure reproducible procedure and help balance tubes.
  It is advisable to avoid watering plants on the day of the experiment.
2 Gently soak leaves in a beaker with distilled water. Repeat the wash three times in separate beakers to remove any soil or dust residue.
3 Dry the leaves by laying them out on a paper towel and gently patting them dry.
4 Once dried, arrange three or four medium‐sized leaves, or two or three large leaves, on top of each other in the same orientation, with veins facing upward. Place leaves into 100‐ or 60‐ml syringes, depending on the size of the leaves and the syringe availability, with the bottoms of the leaves facing the syringe hub.
  Avoid exceeding this quantity of leaves to prevent potential damage during the vacuum infiltration process.
5 Add VIB buffer (90 ml for a 100‐ml syringe or 50 ml for a 60‐ml syringe) and connect the plunger to the syringe. Turn the syringe upside down to remove air bubbles and ensure that the buffer comes into close contact with all the leaves.
  Avoid closing the syringe hub with your finger while you are attaching the plunger, as this could lead to pressure buildup inside the syringe barrel and potentially damage the leaves.
6 Take the air out of the syringe barrel and then place a piece of Parafilm on top of the syringe to block air entry. Use your finger for extra support if needed.
7 Generate a vacuum by pulling the plunger, and then pause for 5 s before gradually returning it. Remove any air bubbles that may appear. Typically, three rounds of vacuum treatment are adequate, though a fourth round may be necessary for larger leaves. This procedure ensures full infiltration of the leaves, with the infiltrated spaces visibly appearing darker.
  It is essential to stay within this limit to avoid damage to the leaves. Also, be careful not to let the plunger fall back automatically, as this could damage the leaves.
8 Remove the buffer from the syringe by detaching the plunger and either pouring it out or allowing the buffer to exit the syringe naturally.
  The buffer can be reused for three or four vacuum treatment cycles, but it should be discarded if any green color is noticed.
9 Remove the leaves by reversing and tapping the syringe on the bench, and then place them on a blue roll or paper towel to pat them dry.
  This step is essential to avoid diluting the AWF.
10 Repeat steps 5‐9 until all leaves have been infiltrated and properly dried.
11 Wrap the dried leaves around a 1‐ml syringe. Begin by arranging four medium‐sized leaves or three large leaves in a uniform orientation by stacking them on top of each other. Cut a strip of tape ∼5 cm in length. Position the 1‐ml syringe at one end of the tape so that it is attached at the middle of the syringe barrel. Position the leaves on top of the tape with their stems facing the plunger, wrap them tightly around the syringe, and secure them with the remaining tape. Once securely wrapped, insert the leaf‐covered syringe into a 50‐ml Falcon tube with the syringe plunger facing downward.
  It is crucial to wrap the leaves tightly but without damaging them. Loosely wrapped leaves may become compressed at the bottom of the tubes during centrifugation, leading to contamination of the extracted AWF. Steps up till this point can be visualized in Video 1.

**Video 1:** Vacuum infiltration of *N. benthamiana* leaves and collection of apoplastic fluid. The video shows the leaf infiltration procedure. Leaves should be loosely wrapped around a 1‐ml syringe, secured with tape, and then placed into the syringe with their stems facing the plunger. The syringe is then inserted into a 50‐ml Falcon tube with the plunger facing downward. The video and music were edited using iMovie.

### Isolation of sEVs by differential centrifugation

**Figure 2:**
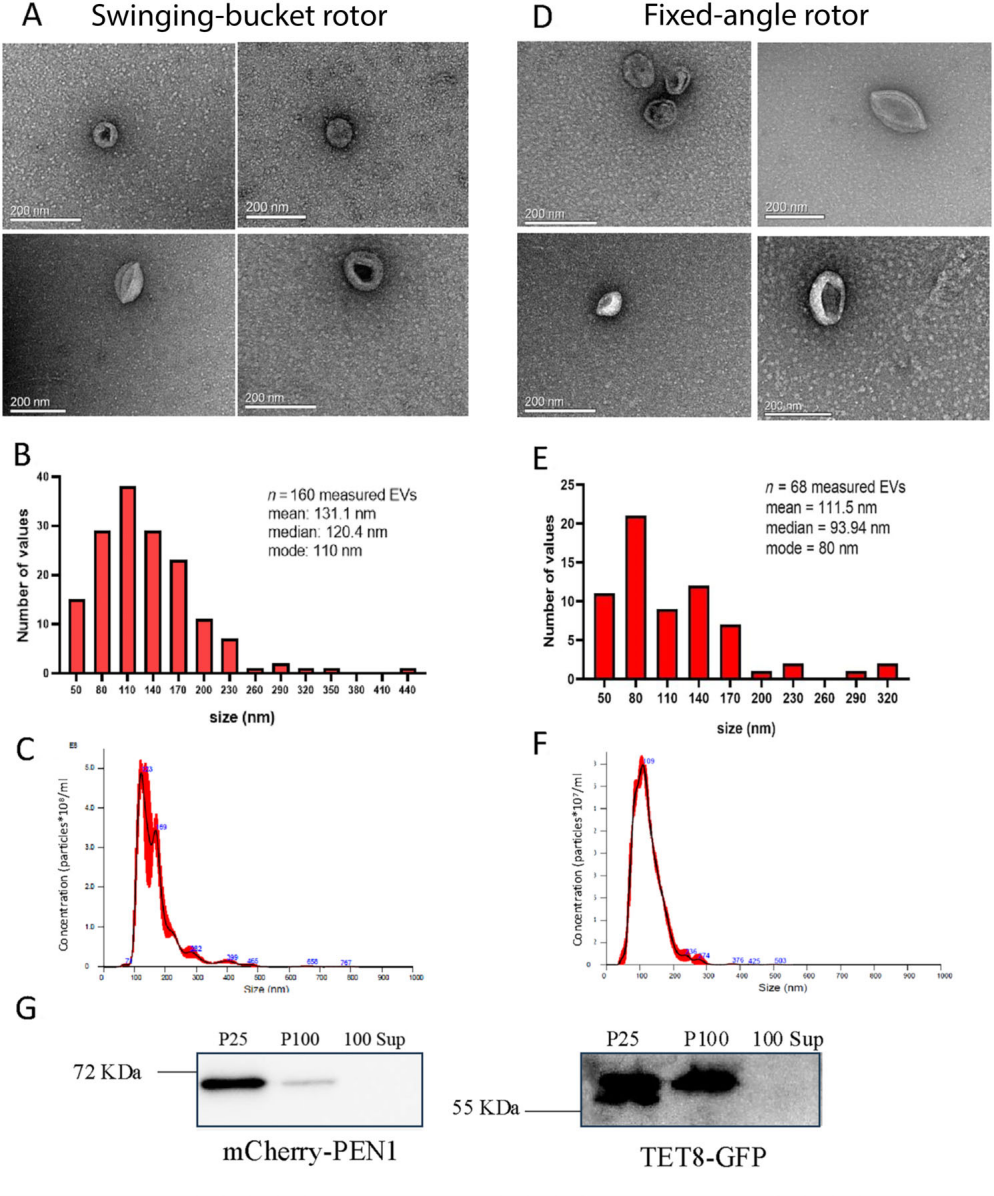
Example of typical results. (**A and D**) Representative transmission electron microscopy (TEM) images of negatively stained EVs (P100) collected with a swinging‐bucket or fixed‐angle rotor and observed at 120 kV with a JEOL JEM‐2100 electron microscope. Scale bars, 200 nm. The size distribution was evaluated using TEM as well as NTA. (**B and E**) Graph representing sEV size measurements from TEM pictures using ImageJ software. *n*, number of sEVs measured. (**C and F**) Representative graphs of size distributions obtained by NTA for preparations made using swinging‐bucket (**C**) and fixed‐angle (**F**) rotors. (**G**) The presence of protein markers was evaluated by western blotting of sEV preparation obtained with fixed‐angle rotors. Agroinfiltration of AtPEN1‐GFP and AtTET8‐mCherry (kindly provided by Dr. Hailing Jin; He et al., 2021) was performed according to Sparkes et al. (2006). At 48 h post infiltration, the leaf tissue was lysed and subjected to ultracentrifugation. 25 μg of protein was loaded into a 10% polyacrylamide gel. The proteins were detected with antibodies against GFP (A‐6455, Thermofisher) and mCherry (26765‐1‐AP, Proteintech). P25 and P100 represent the pellets from 25,000 × *g* and 100,000 × *g* centrifugation, respectively, both using fixed‐angle rotors.

12 Centrifuge tube 15 min at 900 × *g*, 4°C, using a fixed‐angle rotor. If a swinging‐bucket rotor is used, adjust the speed to 600 × *g* for 20 min. In either case, use a reduced acceleration and deceleration set to 3 to ensure minimum damage to the leaves.
  At this stage, we find that using a fixed‐angle rotor is less damaging for leaves. See Critical Parameters for a discussion of the use of fixed‐angle vs. swinging‐bucket rotors.
13 Ensure that the AWF obtained is clear and then transfer it into a fresh tube. The volume recovered will depend on the volume of infiltrated buffer, but 40 ml ± 8 ml is typically expected for 50 g of leaves.
  We recommend discarding any contaminated samples showing a greenish coloration, as this will result in an EV extract contaminated by intracellular vesicles resulting from broken cells. If multiple rounds of centrifugation are required (i.e., leaves are still dark green and therefore contain VIB), keep the AWF tube on ice while performing another round of centrifugation.
14 Centrifuge 10 min at 2000 × *g*, 4°C.
15 Carefully recover the supernatant in a fresh tube and centrifuge 20 min at 10,000 × *g*, 4°C.
16 Transfer the supernatant to a microcentrifuge tube and centrifuge 30 min at 25,000 × *g*, 4°C.
17 Recover the supernatant, transfer to a tube appropriate for high centrifugation speeds (e.g., microcentrifuge tube), and spin 60 min at 100,000 × *g*, 4°C, in a swinging‐bucket or fixed‐angle rotor.
18 Gently remove supernatant, taking care not to disturb the pellet containing the sEVs.
  The pellet is often not visible; therefore, carefully extract the liquid from the top. Before the 100,000 × g spin, it may be helpful to mark the side of the tube where the pellet will be expected. Allow slow acceleration and deceleration, which should take an additional 5 min.
19 Resuspend the pellet in up to 50 μl of PBS or VIB buffer. This will constitute the P100 fraction (P100). Optionally, protease inhibitors can be added at this stage.
20 *Optional*: Centrifuge again for 60 min at 100,000 × *g*, 4°C, to achieve a cleaner product, and resuspend in 50 μl PBS or VIB.
  Please note that although this optional step may enhance purity, it could decrease the total yield.
21 Use immediately or store in aliquots at –80°C.
  If the sample needs to be stored for an extended period, it is optimal to aliquot it to minimize freeze‐thaw cycles. When resuspended in PBS, the sEVs will remain stable for 1 month at –80°C. sEVs purified using this protocol are shown in Figure 2.

## COMMENTARY

### Background Information

Plant EVs serve a variety of functions as versatile carriers of messengers. This exchange is essential for various physiological processes, including plant development, defense responses, and even nutrient exchange (Akuma et al., 2019; Cho et al., 2022). EVs play a crucial role in plant‐microbe interactions by potentially delivering antimicrobial molecules to pathogens, thereby enhancing plant responses to infection (Zhou et al., 2022). Furthermore, plant EVs participate in stress responses, carrying stress signals and protective molecules to help plants cope with harsh environmental conditions (Rutter & Innes, 2017). Finally, plant EVs or plant‐derived nanoparticles (PDNVs) from various sources have been suggested to have beneficial effects on human health (Farley et al., 2022). It is worth noting that EVs are isolated from apoplastic fluid, whereas PDNVs are isolated from whole plants (Pinedo et al., 2021). Therefore, specificities between the two in terms of biological cargo could be discovered in the future.

In general, EV purifications from mammalian sources follow the MISEV guidelines using differential centrifugation followed by various methods such as immunoprecipitation and sucrose gradient. However, because of the variety of plant starting materials, the standardization of procedures for isolating and characterizing plant EVs has progressed at a slower pace than for mammalian EVs (Pinedo et al., 2021).

Isolation of apoplastic sEVs from *N. benthamiana* leaves following a protocol described for *A. thaliana* leaves did not produce satisfactory results and required optimization. Nevertheless, immunoprecipitation and sucrose‐gradient protocols described for *A. thaliana* are adaptable to *N. benthamiana*. Therefore, we focused primarily on the purification of sEVs from apoplastic fluid of *N. benthamiana* leaves by differential centrifugation.

*N. benthamiana* is as a widely used model organism in scientific research, exploring topics ranging from intracellular material transport to host‐pathogen interactions. Its unique ability to act as a “biofactory” for producing human proteins and biologically active substances makes it highly attractive for pharmaceutical applications, particularly evident in its role during the COVID‐19 pandemic, where it contributed significantly to vaccine development (England et al., 2023). As a result, understanding the mechanisms by which *N. benthamiana* EVs contribute to plant growth and defense holds promise for advances in both agriculture and biomedicine.

### Critical Parameters and Troubleshooting

Several critical parameters should be considered to ensure the protocol is successfully performed.

#### Amount of starting material

For optimal EV purification, a minimum of 50 g of tissue is needed, yielding ∼1‐3 × 10^9^ particles/ml in 50 μl, depending on conditions such as rotors. Protein quantification can be performed using the MicroBCA protein assay kit (ThermoFisher) or nanoparticle concentration measured with NTA. It is highly recommended that EV purification be performed on the same day that leaves are harvested. If larger quantities of leaves are used, it is advisable to keep isolated AWF on ice until all AWF is collected. AWF can then be combined before commencing centrifugation steps.

#### Quality of buffers

The quality of the buffer directly impacts the enrichment of sEVs. Phosphate‐buffered saline (PBS) can be prepared ahead using high‐quality double‐distilled water, autoclaved, and stored at 4°C for up to 6 months. However, we recommend always preparing PBS and VIB fresh and autoclaved or filter sterilized. This is most important when resuspending EV pellet to prevent contamination by undesired nanoparticles and avoid having any particles that might be introduced by the buffer itself rather than from the plants. It is also essential to keep VIB cold during subsequent stages of sEV isolation. The quality of prepared buffers may be tested using nanoparticle tracking analysis (NTA) and transmission electron microscopy (TEM) as negative controls to ensure the quality of the preparation and confirm that any observed nanoparticles originate from plant material rather than buffer components. When resuspending pellets, it is crucial to avoid using the buffer used in isolating AWF; instead, clean, unused VIB buffer should be employed.

#### Collection of apoplastic fluid

The critical steps for collecting AWF have been detailed in previous studies (Chen et al., 2022; O'Leary et al., 2014). Substituting the filtration step with a centrifugation step at 25,000 × *g* has not only improved purity but also reduced the overall cost of the process. The 25,000 × *g* centrifugation is crucial for effectively removing contamination by organelles such as chloroplasts and mitochondria, resulting from potential mild cell breakage induced during the earlier vacuum infiltration. The resuspended sEVs after the final 100,000 × *g* centrifugation should appear completely clear. If a greenish color is observed, it is advisable to discard the preparation, as this indicates the presence of broken material that could contaminate the purified sEVs.

#### Centrifugation rotor: Swinging‐bucket or fixed‐angle rotor

The type of rotor available in the laboratory (whether swinging‐bucket or fixed‐angle) is not always flexible. To assess this, we compared EVs prepared using each type of rotor for the final centrifugation at 100,000 × *g*. We noted that both preparations led to comparable EV integrity and diameter sizes, as measured by NTA and TEM for P100: 123 nm ± 6.5 nm (NTA) and 120.4 ± 4.8 nm for swinging‐bucket rotor vs. 109 nm ± 4.1 nm (NTA) and 93.9 ± 7.3 nm (TEM) for fixed‐angle rotors (Fig. 2). However, the quality (presence or absence of aggregates) and amount of EVs will vary depending on which rotors are used during the procedure. Therefore, operators should be mindful of these differences when selecting rotors and should tailor their experiments accordingly, depending on the desired outcome (yield vs. quality, fixed‐angle vs. swinging‐bucket). These results were similar to those previously observed for mammalian EV preparation (Cvjetkovic et al., 2014). Regardless of the type of rotor used, it is advisable to follow EV isolation using differential centrifugation with additional purification steps, such as density gradients or immunoprecipitation. These approaches help separate EV subpopulations based on their density or specific surface markers, allowing for more targeted investigations into their biological functions and potential applications.

See Table 1 for a list of possible problems, causes, and solutions with this protocol.

**Table 1:** Troubleshooting Guide

Problem	Possible cause	Solution
Poor yield	Plant age and/or poor quality or quantity of starting material	Use plants of the proper age and in good shape (e.g., not stressed, not flowering), or increase tissue quantity.
	Pellet recovery	Improve resuspension of the pellet by pipetting up and down more times to allow efficient pellet recovery.
	Poor recovery of apoplastic fluid (AWF)	It is advisable to measure the weight of the leaf both before and after infiltration to accurately calculate the amount of buffer infiltrated. Ideally, the calculated volume should align closely with the volume extracted after the 15 min, 900 × *g* centrifugation. Typically, 1 g of tissue is expected to yield ∼0.8 ml ± 0.2 ml AWF.
Contamination	AWF contaminated by the vacuum or centrifugation procedure	Repeat the procedure, being careful to treat the leaves more gently. Avoid using contaminated AWF.
Green AWF or EV preparation	Contamination by chloroplasts	Discard preparation and repeat the procedure, being careful to treat the leaves more gently.
EV aggregates	Ultracentrifugation is known to cause EV aggregation, and this increases with fixed‐angle rotors	1. Use a swinging‐bucket rotor if possible. 2. Dilute the AWF 1:2 with VIB buffer just before the 100,000 × *g* spin. 3. Add trehalose (10 mM) just before the 100,000 × *g* spin. Ensure that trehalose does not interfere with any downstream application.

### Understanding Results

To assess the quality and purity of the preparation, sEVs should be analyzed through NTA, western blotting, and/or TEM, and ideally all three analyses should be performed for each preparation. Isolation of apoplastic sEVs from *A. thaliana* is somewhat different from the *N. benthamiana* procedure, which requires additional steps. Therefore, it is assumed that slight amendments to the presented protocol will be required to adjust for different plant materials. The TEM results obtained with this protocol will give typical cup‐shaped vesicles of 50 to 200 nm and with a clean background (Fig. 2A and 2D). The size of the isolated particles can be determined using NTA or TEM; NTA is a quicker option, but TEM analysis offers additional insights into the integrity of the isolated EVs (Fig. 2B and 2C). Finally, western blot analysis can confirm the presence and enrichment of sEV subtypes in the final sample. Given the lack of a specific antibody targeting TET8 or PEN1 protein for *N. benthamiana* plants, an agroinfiltrated plant expressing TET8‐GFP or mCherry‐PEN1 was used and protein markers were detected with GFP and m‐Cherry antibodies. It is worth noting that some antibodies raised against *A. thaliana* proteins cross‐react with *N. benthamiana* proteins (not shown).

### Time Considerations

Time is critical when isolating extracellular vesicles (EVs) from plant leaves. To ensure sample integrity, each step, from leaf harvesting to EV purification, must be completed quickly. To speed up the process, two people may need to work together, particularly during the steps from leaf arrangement to leaf infiltration. However, if one person is working alone, cold VIB buffer is essential in slowing leaf degradation and extending the processing time window. Furthermore, the leaf infiltration step could be optimized to include a larger number of leaves to be infiltrated; however, it is important to maintain a balance between efficiency and gentle treatment to ensure EV quality and purity. Depending on the quantity of initial material, this protocol can be completed in 5 to 6 hr.

### Author Contributions

**Mahmoud Eldahshoury**: Conceptualization; formal analysis; investigation; methodology; validation; writing—original draft; writing—review and editing. **Konstantina Katsarou**: Conceptualization; formal analysis; investigation; methodology; validation; visualization; writing—original draft; writing—review and editing. **Joshua Farley**: Investigation; methodology. **Kriton Kalantidis**: Conceptualization; data curation; funding acquisition; project administration; resources; supervision; validation; writing—review and editing. **Carine de Marcos Lousa**: Conceptualization; data curation; formal analysis; funding acquisition; methodology; project administration; resources; supervision; validation; writing—original draft; writing—review and editing.

### Conflict of Interest

The authors declare no conflict of interest.

## Data Availability

Original files for TEM, NTA, and western blot data are available upon request.
